# New parameter for QRS complex low voltage in chagasic cardiomyopathy: the ADOC index

**DOI:** 10.1590/0037-8682-0216-2024

**Published:** 2025-02-07

**Authors:** Ana Gabriela Miranda Barbosa, José Antonio da Silva, Ellany Gurgel Cosme do Nascimento, Mariana de Moura Góes, Antônio Almeida dos Santos, Rodrigo Alves de Melo, Remerson Russel Martins, Thales Allyrio Araújo de Medeiros Fernandes, Micássio Fernandes de Andrade, Cléber de Mesquita Andrade

**Affiliations:** 1 Universidade do Estado do Rio Grande do Norte, Departamento de Ciências Biomédicas, Mossoró, RN, Brasil.; 2 Universidade Federal Rural do Semi-Árido, Mossoró, RN, Brasil.

**Keywords:** Chagas Disease, Chagas Cardiomyopathy, Electrocardiography, Low voltage of QRS complex, Mean amplitude of QRS complexes

## Abstract

**Background:**

Low QRS complex voltage is an important predictor of death in Chagas disease. However, the parameters applied to the low-voltage classification were described by the Minnesota Code and not specifically for Chagas disease. This study aimed to analyze low QRS voltage by determining the ADOC index and averages in the frontal and horizontal electrocardiographic planes, establishing possible clinical implications.

**Methods:**

A cross-sectional study of patients with Chagas disease was performed using the Mann-Whitney U test and Spearman’s correlation. The amplitudes of each QRS were analyzed, and the sum of the DII and V5 derivations of the ADOC index and the arithmetic means of the QRS complexes in the frontal and horizontal planes were determined.

**Results:**

The ADOC index was correlated with the highest risk of stroke and death according to the Rassi score. The ADOC index (p=0.046) and mean mQRS were inversely proportional to the Rassi risk of death score (p=0.038). The ADOC index proved to be more sensitive (75.0%) and accurate (67.4%) in identifying patients at elevated death risk using the Rassi score. Finally, a positive correlation was observed between the QRSFm and QRSHm indicators and ADOC index (r=0.590 and r=0.857, respectively).

**Discussion:**

The ADOC index and mean of the QRS complexes are possible tools correlated with the Rassi score and risk of stroke in patients with Chagas disease.

## INTRODUCTION

Cardiac form is the most severe clinical presentation of chronic Chagas disease (CD). Incessant low-intensity focal fibrosing myocarditis, resulting from persistent *Trypanosoma cruzi* infection, is linked to an inflammatory reaction by immune mechanisms^1^. It develops in 20-30% of individuals and leads to conduction system abnormalities, bradyarrhythmias/tachyarrhythmias, apical aneurysms, thromboembolism, congestive heart failure, ischemic stroke, and sudden death, the last one being a result of complex ventricular arrhythmias^2^ ^-^ ^5^.

Considering the high degree of morbidity and mortality in chronic Chagas’ heart disease and the relevance of some examinations in diagnosis and predicting prognosis in CD, the Rassi score developed in 2006 aims to quantify the risk of death in 10 years based on six criteria, including the low voltage of the QRS complex^6^.

The plausibility of using low-voltage QRS complexes to assist with the continuation of CD diagnostic investigations has been demonstrated previously, with a proposed and validated tool to support these decisions identifying 13 diagnostic predictive factors, among which a low QRS complex voltage has been shown to contribute with CD diagnostic investigations^7^.

The Minnesota Code^8^, published in 1960, has been designated for studies of coronary diseases; however, it does not address the various and complex forms of arrhythmias and conduction defects characteristic of Chronic Chagas Cardiomyopathy (CCC). It is the reference used for studies in CD, including parameters for determining low QRS complex voltage. Other studies have been conducted, such as the adapted Minnesota Code, to adapt to the characteristics of trypanosomiasis; however, there have been no updates regarding the parameters for the low voltage of the QRS complex^9^.

Low-voltage determination of the current QRS complex consists of the amplitude of the complexes in the frontal plane below 5 mm and the horizontal plane below 10 mm^8^. This criterion must be reconsidered for better representation of the chagasic population. Instead of requiring all electrocardiographic leads to necessarily conform to the Minnesota Code, determining the average or sum of the voltages from the most representative leads in each plane (frontal and horizontal) may more appropriately classify low voltages in CD.

Leads such as the DII (frontal plane) and V5 (horizontal plane), which capture electrical activity to the left of the heart, are normally located on the cardiac axis and consequently tend to have higher voltages. Therefore, if low amplitudes were detected from the QRS assessment of these leads, determining the low voltage would most likely be more accurate. Similarly, by establishing the average of the complexes, greater sensitivity could be achieved.

Because of the importance of assessing the QRS in CD, we aimed to analyze a possible clinical application of its low voltage by determining the averages in the frontal and horizontal planes, correlating them with the prognosis and changes in complementary examinations during patient follow-up, and seeking to establish new determining variables of the low amplitude of the QRS complex, directed towards CD, preferentially involving the frontal and horizontal planes.

## METHODS

This was a descriptive, observational, and cross-sectional study, in which data were obtained from a population of 185 patients, comprising 95 women and 90 men, aged 22-78 years, followed by the *Ambulatório de Doença de Chagas* (ADOC) of the State University of Rio Grande do Norte, from the municipalities of the Western mesoregion of the Rio Grande do Norte State.

The patients were seropositive based on at least two of three different serological methods^10^ ^-^ ^11^ (Enzyme-Linked Immunosorbent Assay, indirect immunofluorescence, and/or indirect hemagglutination), presenting a defined clinical form^12^ ^-^ ^14^ (indeterminate, cardiac, digestive, or cardiodigestive).

Patients who did not have all the information necessary for analysis of the variables in their medical records and those who failed to undergo complementary examinations to define the clinical form of the disease were excluded from the study.

### Determination of the clinical form, risk of death by Rassi score, and risk of stroke

To establish the clinical form, in addition to the clinical history, performing complementary tests, namely electrocardiography, simple chest radiography, and contrast-enhanced esophageal and colonic radiography, was necessary. After performing transthoracic echocardiography and dynamic electrocardiography (Holter system), the risk of death score was applied (Rassi’s risk of death)^6^, classifying patients as high, moderate, or minimal risk depending on their scores, as well as the score for stroke risk^15^. Rassi’s risk of death was calculated only for patients with cardiac involvement related to CD^6^. 

### Determination of the averages of the frontal and horizontal QRS and ADOC Index

To advance this study, the voltage amplitudes of each QRS complex in their respective leads were measured using calibrated paper and a millimeter ruler. Frontal (QRSFm) and horizontal (QRSHm) averages were determined using arithmetic means and the ADOC index (the sum of the QRS complex voltages in leads DII and V5).

QRSFm=DI+DII+DIII+aVF+aVL+aVR6QRSHm=V1+V2+V3+V4+V5+V66ADOC Index =∑DII+V5

### Study variables

The dependent variables were the voltage amplitudes of QRSFm and QRSHm as well as the ADOC index, and the independent variables comprised sex, risk of death by Rassi score, cardiomegaly, risk of stroke, ventricular premature complex (VPC), right bundle branch block (RBBB), left anterior fascicular block (LAFB), and presence of thrombus on transthoracic echocardiogram.

From the medical records of the patients, the results of complementary examinations of a simple chest radiograph, contrast-enhanced esophagus and colon, electrocardiogram, Holter system, and transthoracic echocardiogram were collected.

The cardiothoracic index (CTI) was measured using a posteroanterior chest radiograph, with cardiomegaly defined as a CTI >0.5^16^. Transthoracic echocardiography was performed by a single observer, an experienced cardiologist, using conventional views and their variations to identify segmental contractility abnormalities, including the presence of ventricular aneurysms and to highlight the presence of images suggestive of intracardiac thrombi. Linear measurements of the cardiac chambers were performed according to the recommendations of the American Society of Echocardiography^17^.

### Study limitations

The less frequent electrocardiographic alterations in this sample of participants could provide more reliable results regarding their clinical outcomes based on the analysis of a larger population sample.

As this was a cross-sectional observational study, analyzing the achievement of clinical outcomes related to electrocardiographic findings in this sample of participants was not possible. Therefore, longitudinal studies and/or randomized clinical trials are warranted^18^.

### Statistical and ethical analysis

The data were analyzed using IBM SPSS Statistics for Windows 20. The Mann-Whitney U test was used for independent samples, and the Spearman correlation coefficient was used to analyze the relationships between continuous quantitative variables of the indicators. Non-parametric tests were used because of the non-normal distribution of the data.

A statistical significance level of 5% was considered when defining type I or alpha error. To calculate the power of the test (type II or beta error), the results calculated for the predictive values were considered.

Sensitivity, specificity, positive predictive value, negative predictive value, and accuracy were used to calculate the performance characteristics of the tests. To this end, we considered as truly positive for the calculation the presence of high Rassi risk of death and high risk of stroke associated with the ADOC index amplitude values (≤1.6 mV), QRSHm (≤1.0 mV), and QRSFm (≤0.5 mV). The low/intermediate Rassi risk of death and low/moderate risk of stroke were considered truly negative for the calculation and were associated with the amplitude values of the ADOC index (>1.6 mV), QRSHm (>1.0 mV), and QRSFm (>0.5 mV).

The study was approved by the Research Ethics Committee of the State University of Rio Grande do Norte, under protocol number 1.160.553 and CAAE 43783915.3.0000.5294 on 07/21/2015, following the requirements of Brazilian National Resolution number 466/2012, which considers the ethical principles of the Declaration of Helsinki. A total of 185 participants read and signed an Informed Consent Form to participate in the study.

## RESULTS

Among the participants, 51.4% (95/185) were women, 20.5% (38/185) had cardiomegaly on plain chest radiography, and 2.2% (4/185) had a thrombus on transthoracic echocardiography. The prevalence of RBBB was 14.6% (27/185), that of LAFB was 10.3% (19/185), and that of both RBBB and LAFB was 5.4% (10/185). Table 1 provides additional information on the independent variables.

**TABLE 1:** Descriptive analysis of the risk of death, risk of stroke, and frequency of premature ventricular complexes (PVC) on 24-hour Holter in patients with Chagas disease.

Variables	n=185	%
**24-hour Holter PVC frequency**		
High frequency	24	13.0
Low frequency	47	25.4
Moderate frequency	09	4.9
Absent	23	12.4
Not determined	82	44.3
**Rassi's risk of death**		
Low	55	29.7
Moderate	25	13.5
High	12	6.5
Not applicable	93	50.3
**Stroke risk**		
Low	151	81.6
Moderate	10	5.4
High	21	11.4
Not determined	03	1.6

**n:** number of patients; **%:** Percentage; **CVA:** ischemic stroke; **PVC:** premature ventricular complex.

The QRSHm and ADOC index values between men and women were statistically significant, with the mean ADOC index being lower in women than in men. The QRSHm value in women was below the considered minimum limit^8^ and the QRSHm in men was above the limit. The QRSFm was not statistically significant (Table 2).

**TABLE 2:** Mean value and p-value of the Mann-Whitney U Test of the ADOC Index, QRSFm, and QRSHm by sex, Rassi’s risk of death, frequency of PVC, RBBB, LAFB, RBBB and LAFB, thrombus, and cardiomegaly in patients with Chagas disease.

Variables	ADOC index		QRSFm		QRSHm	
	Average (SD)	P	Average (SD)	P	Average (SD)	P
**Sex**						
Female (n=90)	1.880 (0.633)	0.001	0.622 (0.205)	0.198	0.991 (0.273)	<0.001
Male (n=95)	2.203 (0.677)		0.591 (0.203)		1.237 (0.304)	
**Risk of death**						
Low/Intermediate (n=80)	1.985 (0.710)	0.046	0.620 (0.226)	0.038	1.087 (0.348)	0.369
High (n=12)	1.608 (0.620)		0.492 (0.176)		0.957 (0.272)	
**PVC frequency**						
Low (n=47)	2.174 (0.703)	<0.001	0.609 (0.235)	0.697	1.188 (0.334)	0.004
High (n=24)	1.546 (0.486)		0.579 (0.229)		0.946 (0.230)	
**RBBB AND LAFB**						
Yes (n=10)	1.870 (0.862)	0.222	0.637 (0.240)	0.782	0.979 (0.449)	0.038
No (n=175)	2.056 (0.664)		0.604 (0.203)		1.126 (0.304)	
**Thrombus**						
Yes (n=4)	1.225 (0.597)	0.016	0.485 (0.308)	0.357	0.802 (0.239)	0.036
No (n=181)	2.066 (0.668)		0.608 (0.201)		1.125 (0.313)	
**Cardiomegaly**						
Yes (n=38)	1.871 (0.596)	0.063	0.636 (0.251)	0.619	1.070 (0.288)	0.325
No (n=145)	2.091 (0.690)		0.596 (0.189)		1.130 (0.320)	
**RBBB**						
Yes (n=27)	1.915 (0.712)	0.221	0.610 (0.226)	0.849	1.034 (0.363)	0.059
No (n=158)	2.068 (0.667)		0.605 (0.201)		1.132 (0.304)	
**LAFB**						
Yes (n=19)	1.863 (0.780)	0.123	0.642 (0.233)	0.513	1.034 (0.406)	0.063
No (n=166)	2.067 (0.660)		0.602 (0.201)		1.127 (0.302)	

**n:** number of patients; **%:** Percentage; **CVA:** ischemic stroke; **PVC:** premature ventricular complex; **QRSFm:** frontal QRS complex amplitude; **QRSHm:** horizontal QRS complex amplitude; **RBBB:** right bundle branch block; **LAFB:** left anterior superior divisional block.

The mean QRSFm and ADOC index were inversely proportional; the smaller the score, the higher the Rassi score^6^, that is, a high risk of death in 10 years, which was also statistically significant. The QRSHm values were not statistically significant (Table 2). The risk of stroke and frequency of VPC showed a statistically significant statistical association when compared with the QRSHm values and ADOC index. No statistically significant association was observed between QRSFm values and any other variables (Table 2).

The ADOC index and QRSHm values showed a statistical association with the presence of thrombus on the transthoracic echocardiogram, with lower amplitude in both; the QRSHm of patients with thrombus was below 1 mV, a current criterion for low voltage^8^. Only QRSHm values showed a statistically significant association with the presence of RBBB and LAFB. No statistically significant associations were established between QRSFm, QRSHm, and ADOC index and cardiomegaly, RBBB, and LAFB variables alone (Table 2).

**FIGURE 1:**
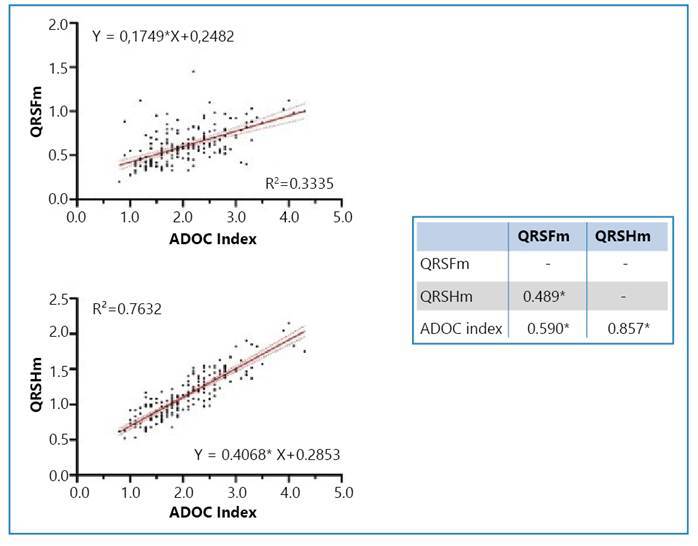
Spearman correlation and logistic regression between QRSFm values, QRSHm ADOC index, and risk of stroke in patients with Chagas disease (n=185). Own (2019). * Correlation is significant at the 0.01 level (bilateral). QRSFm, frontal QRS complex amplitude; QRSHm, horizontal QRS complex amplitude.

Spearman’s correlation was positive between the QRSFm and QRSHm indicators and the ADOC index (Figure 1).

The ADOC index proved to be more sensitive, with a higher negative predictive value and greater accuracy than QRSFm and QRSHm for identifying patients at high risk of death using the Rassi score. For patients at a high risk of stroke, the ADOC index proved to be more specific and had a greater negative predictive value and accuracy than the QRSFm and QRSHm (Table 3).

**TABLE 3:** Values for sensitivity, specificity, positive and negative predictive value, and accuracy of the ADOC Index, QRSHm, and QRSFm concerning the Rassi’s risk of death and the risk of stroke (n=185).

	Sensitivity (%)	Specificity (%)	PPV (%)	NPV (%)	Accuracy (%)
**Rassi's Risk of death** ^6^					
ADOC index	75.0	66.2	25.0	94.6	67.4
QRSHm	58.3	67.5	21.2	91.5	66.3
QRSFm	50.0	47.5	12.5	86.4	47.8
**Stroke risk**					
ADOC index	57.1	73.3	21.8	92.9	71.4
QRSHm	61.9	60.9	17.1	92.4	60.9
QRSFm	42.9	68.3	15.0	90.2	65.4

**QRSFm:** frontal QRS complex amplitude; **QRSHm:** horizontal QRS complex amplitude; **PPV:** Positive Predictive Value; NPV: Negative Predictive Value.

**FIGURE 2:**
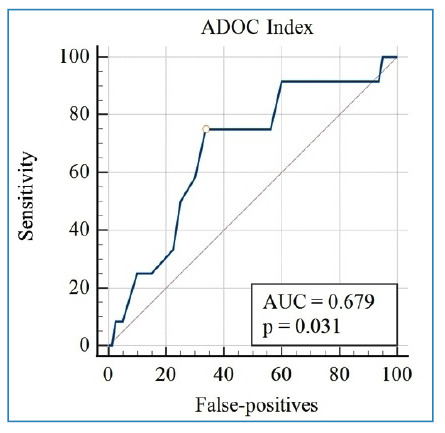
ROC curve of the ADOC index. **AUC:** area under the curve.

Figure 2 shows the behavior of the ADOC index in its projection of the ROC curve, where the area under the curve was 0.679 and the Youden index was 1.6, which was considered the cutoff point in this study.

## DISCUSSION

A low QRS complex voltage amplitude in patients with CD of both sexes is associated with a worse prognosis, as identified by the risk of death using the Rassi score^6^. Appreciating the correlation between sex and the QRSHm means and ADOC index, the Cornell criterion^19^ ^-^ ^20^ corroborates the results, considering that left ventricular overload is influenced by sex. This criterion, when compared with other criteria for left ventricular overload, was the most sensitive for diagnosis in women^20^.

Cornell determined a maximum voltage of 28 mm for men and 20 mm for women (resulting from the sum of the amplitude of the R wave of lead aVL with the S wave of precordial lead V3)^21^, possibly considering a lower cardiac mass index among women. In this study, a QRSHm below 1 mV (10 mm) was obtained, which fit the current low voltage criteria: below 0.5 mV (5 mm) for electrocardiographic leads in the frontal plane and below 1 mV (10 mm) in the horizontal plane^8^ ^,^ ^17^.

Despite not meeting the current low-voltage criterion, the average ADOC index was also lower in women, which corresponded to expectations, considering that the DII and V5 leads tend to have the greatest amplitudes in their respective electrocardiographic planes, mainly representing the left ventricular mass. However, an average above 1 mV does not exclude the possibility that the patient has a low QRS voltage, but demonstrates that this parameter has a cutoff point different from current standards, having shown a relevant sensitivity concerning the other methods used in this study.

In an inverse proportion, patients at high risk of death showed lower QRSFm means, below 0.5 mV, which is considered the lower limit for low voltage by the Minnesota code^8^. This result is in line with the predictor risk score for CD mortality, in which low voltage of the QRS complex accounts for two points, among other prognostic factors such as heart failure functional classification NYHA (New York Heart Association) III and IV, cardiomegaly, non-sustained ventricular tachycardia, ventricular dysfunction on echocardiography, and male sex^6^.

The ADOC index, concerning the risk of death, presents lower averages in patients with high Rassi score^6^ ^,^ which validates its use as a practical method for assessing the potential risk in patients with CC. This corroborates the previous hypothesis that the sum (∑DII+V5) has greater sensitivity in determining the low amplitude and therefore greater risk of mortality in these patients. 

Similar to the risk of death, the risk of stroke^15^ is inversely proportional to the QRSHm means and ADOC index. Therefore, patients with lower voltages in the QRSHm may be at a higher risk of ischemic cerebrovascular events. Therefore, because stroke is one of the main mechanisms of death in CD, together with sudden death and congestive heart failure^5^, this result is similar to that of the risk of death. According to a previous study, individuals infected with *T. cruzi* exhibit higher rates of cardioembolic stroke when compared with the control group without CCC (56% vs. 9%)^22^.

When considering the high and low frequency (above or below 400 episodes per day) of VPC (beat originating in the ventricle, early, with a post-extrasystolic pause, when cycling the RR interval)^23^, a higher incidence of high-frequency VPC in patients with greater underlying myocardial damage, represented by lower means of the QRSHm and ADOC index, was observed^24^. Cardiac autonomic dysfunction, present early in CCC, predisposes patients to malignant arrhythmias and sudden death^5^ ^,^ ^25^ ^-^ ^26^. For example, even patients with an undetermined clinical form may be at risk of developing ventricular arrhythmia when exposed to dynamic electrocardiography (Holter system)^27^. 

The presence and frequency of arrhythmia normally correlate with the degree of ventricular dysfunction; however, in CCC there is the “isolated arrhythmogenic form” of the disease, which distinguishes cardiopathy due to CD from coronary disease with ventricular dysfunction^1^. Other fundamental mechanisms for the onset of severe ventricular arrhythmias include the presence of regional fibrosis, especially in the inferolateral and apical LV regions, and the formation of macro-reentry circuits, with sudden death being one of the main consequences^5^ ^,^ ^28^ ^-^ ^31^.

The persistent tissue inflammatory activity in CCC culminates in important morphophysiological changes owing to multiple cumulative lesions^11^. In this context, cardiomegaly is expected to be associated with low voltage in the average QRS complex amplitudes in the frontal and/or horizontal planes and/or the ADOC index, considering the increased myocardial fibrosis resulting from chronic and persistent myocarditis in these patients^32^ ^-^ ^34^, this could potentially explain the occurrence of low QRS complex voltage in CCC^35^. However, contrary to expectations, the mean amplitudes of the frontal and horizontal QRS complexes and the ADOC index were not statistically significantly associated with the presence of cardiomegaly in the patients in this study. This result may be explained by the low sensitivity of simple chest radiography in identifying this cardiac finding^36^ ^-^ ^38^. 

This result may be explained by the low accuracy of simple chest radiography in determining left ventricular enlargement, as demonstrated in previous studies comparing the CTI and left ventricular enlargement via transthoracic echocardiography, which provides a more precise analysis of the linear dimensions of the cardiac chambers. The unreliability of CTI measurement by simple chest radiography may be influenced by several factors, such as the radiographic technique, thoracic abnormalities such as scoliosis, lordosis, pectus excavatum, lung size, degree of pulmonary inspiration^39^, and the fact that CTI may also encompass atrial dilation^40^.

Notably, previous studies have demonstrated a significant presence of thromboembolic manifestations of cardiac origin associated with CD, present in 44-73% of necropsies of patients with CCC^41^ ^-^ ^42^ including, documented evidence that the frequency of these events is higher in CCC than in other dilated cardiomyopathies^43^. Thrombus formation is linked to predisposing cardiac morphophysiological alterations, such as cavity dilation and ventricular regional dyskinesia, allowing greater blood stasis and cardioembolic manifestations^1^ ^,^ ^11^ ^,^ ^44^ ^-^ ^46^.

However, the sample size of patients with thrombotic formation in this study was too small to ensure high reliability in the data cross-analysis. Despite the small sample size, this finding may reflect a spurious association. Therefore, a low QRS complex voltage would not indicate the presence of a thrombus but rather the presence of areas of akinesia, dyskinesia, or ventricular aneurysm secondary to the fibrosing myocarditis characteristic of the disease^35^.

The most common electrocardiogram (ECG) abnormalities in CCC are RBBB, LAFB, VPC, ST-T changes, abnormal Q waves, and a low QRS voltage. The combination of RBBB and LAFB is typical in CCC^4^, and their presence in electrocardiographic tracings is associated with a higher frequency of VPC and sudden death^47^.

According to a study, in a group of 180 patients, among whom 21 died, a much higher incidence of electrocardiographic alterations was identified in the lethal group than in the non-lethal group (85.7% and 37.7%, respectively), and low QRS voltage (15 of 21 patients) predominated in lethal cases, and only two cases that presented complete RBBB died. Therefore, the importance of these two electrocardiographic findings in the prognosis of patients with CD can be deduced^6^ ^,^ ^48^.

Given the identification criteria for RBBB and LAFB, there is no correlation between the presence of an RBBB or LAFB and the voltage of the QRS complexes in the literature^49^. However, in patients who simultaneously underwent RBBB and LAFB, a lower QRSHm^8^ was observed. Therefore, it can be presumed that the presence of simultaneous RBBB and LAFB (a frequent finding in CCC), but not isolated (RBBB or LAFB), may be related to a lower voltage in the horizontal plane, configuring a possible new criterion for identifying these findings on electrocardiography.

Regarding the performance results of the ADOC index, the sensitivity for Rassi’s risk of death was 75.0% and stroke risk was 57.1%, presenting a much better performance when comparing the sensitivity of the indices used for the quantification of left ventricular overload, such as Sokolow-Lyon-Rappaport and Cornell, which presented sensitivities of 22.0% and 42.0%, respectively^50^, although they remain widely used in the field of cardiology because of their excellent cost-benefit ratio.

Low QRS complex voltage is a characteristic finding with prognostic significance for CD, as demonstrated by the Rassi score^6^. Analyzing its clinical and prognostic implications as well as its contribution to predicting the risk of death can be an additional tool in medical practice.

## CONCLUSIONS

The average of the QRS complexes in the frontal and horizontal planes and the ADOC index are possible tools for investigating clinical outcomes such as the risk of death and stroke. In addition, these averages are linked to characteristic findings in fundamental tests for the follow-up of chronic patients with CD, such as high frequency of VPC on 24-hour Holter, RBBB associated with LAFB on electrocardiography, and thrombus on transthoracic echocardiography. Therefore, it can be considered a warning factor for the development of these complications in patients with CD, helping in their management and therapeutic decision-making.
